# Development of an Immunofluorescence Assay Module for Determination of the Mycotoxin Zearalenone in Water

**DOI:** 10.3390/toxins13030182

**Published:** 2021-03-02

**Authors:** Borbála Gémes, Eszter Takács, Patrik Gádoros, Attila Barócsi, László Kocsányi, Sándor Lenk, Attila Csákányi, Szabolcs Kautny, László Domján, Gábor Szarvas, Nóra Adányi, Alexei Nabok, Mária Mörtl, András Székács

**Affiliations:** 1 Agro-Environmental Research Centre, Institute of Environmental Sciences, Hungarian University of Agriculture and Life Sciences, Herman O. út 15, H-1022 Budapest, Hungary; gemes.borbala.leticia@uni-mate.hu (B.G.); takacs.eszter84@uni-mate.hu (E.T.); mortl.maria@uni-mate.hu (M.M.); 2 Department of Atomic Physics, Budapest University of Technology and Economics, Műegyetem rkp. 3, H-1111 Budapest, Hungary; gadorosp@eik.bme.hu (P.G.); barocsi@eik.bme.hu (A.B.); kocsanyi@eik.bme.hu (L.K.); lenk@eik.bme.hu (S.L.); 3 Optimal Optik Ltd., Dayka Gábor u. 6/B, H-1118 Budapest, Hungary; attila_csakanyi@optimal-optik.hu (A.C.); szabolcs_kautny@optimal-optik.hu (S.K.); domjan@optimal-optik.hu (L.D.); gabor_szarvas@optimal-optik.hu (G.S.); 4 Food Science Research Centre, Institute of Food Sciences, Hungarian University of Agriculture and Life Sciences, Herman O. út 15, H-1022 Budapest, Hungary; adanyi.nora@uni-mate.hu; 5 Materials and Engineering Research Institute, Sheffield Hallam University, Howard Street, Sheffield S1 1WB, UK; a.nabok@shu.ac.uk

**Keywords:** zearalenone, mycotoxin, competitive immunoassay, fluorescence detection, high-performance liquid chromatography, total internal reflection ellipsometry

## Abstract

Project Aquafluosense is designed to develop prototypes for a fluorescence-based instrumentation setup for in situ measurements of several characteristic parameters of water quality. In the scope of the project an enzyme-linked fluorescent immunoassay (ELFIA) method has been developed for the detection of several environmental xenobiotics, including mycotoxin zearalenone (ZON). ZON, produced by several plant pathogenic *Fusarium* species, has recently been identified as an emerging pollutant in surface water, presenting a hazard to aquatic ecosystems. Due to its physico-chemical properties, detection of ZON at low concentrations in surface water is a challenging task. The 96-well microplate-based fluorescence instrument is capable of detecting ZON in the concentration range of 0.09–400 ng/mL. The sensitivity and accuracy of the analytical method has been demonstrated by a comparative assessment with detection by high-performance liquid chromatography and by total internal reflection ellipsometry. The limit of detection of the method, 0.09 ng/mL, falls in the low range compared to the other reported immunoassays, but the main advantage of this ELFIA method is its efficacy in combined in situ applications for determination of various important water quality parameters detectable by induced fluorimerty—e.g., total organic carbon content, algal density or the level of other organic micropollutants detectable by immunofluorimetry. In addition, the immunofluorescence module can readily be expanded to other target analytes if proper antibodies are available for detection.

## 1. Introduction

Natural mycotoxin contamination has been identified as an emerging problem in agriculture. These toxic secondary metabolites produced by some fungal species are often found in food and feed (especially in grains) and cause high risk for food- and feed-borne intoxication in both humans and livestock [1]. The wide range of their negative effects includes, e.g., genotoxic, cytotoxic, mutagenic, and teratogenic effects [2]. Among all the toxic filamentous fungi species, *Aspergillus*, *Fusarium*, and *Penicillium* are important genera, producing regularly detected and widely studied toxins including aflatoxins, ochratoxin A, deoxynivalenol, T-2 toxin, fumonisin, and zearalenone (ZON) [3]. Mycotoxins found in human urine, indicate the possibility of chronic exposure [4]. In addition, a northward migration of toxicogenic plant pathogenic fungi has been reported assumedly triggered by climate change [5,6,7]. ZON is a frequently occurring mycotoxin, produced by species of the *Fusarium* genus, including *F. graminearum*, *F. culmorum*, *F. semitectum*, *F. cerealis*, and *F. equiseti* [8]. Its most known impact on human health is endocrine disruption: ZON and derivatives trigger estrogen-like effects in mammals causing alteration in hormone-mediated processes, e.g., the production of follicle-stimulating hormone (FSH) and luteinizing hormone (LH), and reductions in the number of Leydig and granulosa cells [9]. The potential health and economic impacts of ZON necessitate its routine monitoring in food and commodities, which have led to the development and validation of analytical methods in recent decades.

### 1.1. Mycotoxins as Pollutants in Surface Waters

In addition to food- and feed-borne intoxication, humans can also be affected through exposures via surface water contamination. Various phytopathogenic fungi, including *Fusarium* species, have been demonstrated to be capable of continuing to produce their secondary metabolites in water [10,11], and this process has been indicated to be a potential route of human exposure to mycotoxins [12]. Numerous investigations have reported the presence and input pathways of the toxin in surface or groundwater [13,14,15,16,17]. Mycotoxins may occur in surface waters by direct fungal contamination, by leaching from infested soil as water runoff from agriculture, by washing out from contaminated agricultural commodities such as cereals, oil, forage, feed etc. [18,19], or by mycotoxin biosynthesis in water by fungi [20,21]. In turn, mycotoxins have been considered as emerging surface water contaminants of diffuse and point source occurrence [22], through which fungal contaminants are considered emerging evidence-based threats for drinking water quality safety regulations [23]. Thus, water contamination by aflatoxins B2 and G2 were detected in water in Southern England at levels of 0.1–1.7 ng/mL [24]; aflatoxins B1 and B2, fumonisin B3, and ochratoxin A were detected at concentrations up to 0.035 ng/mL in the Tagus river Portugal [25]; phytoestrogens and mycotoxins were monitored in agricultural stream basins in the United States in Iowa, with occasional occurrence of deoxynivalenol above 0.1 ng/mL level [26]; aflatoxins B2, B1, and G1, as well as ochratoxin A were detected at levels of no toxicological risk up to 0.0007 ng/mL in bottled water in Portugal [27]; fumonisins were detected at up to 0.048 ng/mL levels in aqueous environmental samples in Poland [28]. Along with other mycotoxins, ZON and its metabolites also appear to be water contaminants. ZON was in found in surface waters, groundwater, and wastewater in Poland at levels up to 0.081 ng/mL, originating from cereal crops [14,15], and ZON, along with the metabolites zearalanone, α-zearalenol, β-zearalenol, α-zearalanol, and β-zearalanol, was detected in surface waters in Brazil at levels up to 4.12 ng/mL [29]. In total, 32 of 159 surface water samples collected in central Illinois have been positively tested, and 10 of them were above limit of quantification (LOQ) with concentrations between 0.002 and 0.006 ng/mL [30]. ZON also has been found in nine samples collected from eight Portuguese rivers and creeks ranging between 0.006 and 0.083 ng/mL [17], and appeared in the drainage water of a *F. graminearum* infected field in Switzerland with higher concentrations in the summer vegetation periods in a two-year field experiment [31]. Lower but detectable (0.002–0.005 ng/mL) concentrations were found in the Tiber river in Italy [32].

Moreover, the appearance of mycotoxins in the aquatic environment can adversely influence entire ecosystems. Thus, *Fusarium* mycotoxins, including ZON, can exert hormonal (estrogenic), hepatotoxic or genotoxic effects on fish [33,34]. Nonetheless, maximal residue levels (MRLs) to ensure compliance with the tolerable daily intake for humans (µg/kg body weight) by the European Union legislation [35] have been set only for food and feed, e.g., MRLs from 20 µg/kg in processed maize-based foods for infants and young children up to 200 µg/kg in unprocessed maize [36]. There is also a commission recommendation for ZON (and other mycotoxins) in products intended as animal feed as recommended by two scientific opinions of the European Food Safety Authority [37,38], but no declared maximum level for drinking water or surface waters have been established yet.

### 1.2. Analytical Methods for Zearalenone Determination

There are numerous well-established methods for quantification of ZON, varying in their technical detail (e.g., sample preparation, principle of the analytical procedure) according to the complexity of the target matrix and other circumstances. Analytical methods for detection include colorimetric and fluorescence-based strategies (e.g., enzyme-linked immunosorbent assay, ELISA), chromatographic methods, and enzyme-linked oligonucleotide assays [39]. Traditional chromatographic separation, e.g., high-performance liquid chromatography (HPLC) [40,41,42], thin layer chromatography (TLC) [43,44,45], and liquid or gas chromatography coupled with mass spectroscopy (LC or GC MS) [46,47,48,49] have low limit of detection (LOD) and limit of quantification (LOQ) values but, usually, due to the complex sample preparation they are time consuming technologies requiring special instrumentation. Immunoanalytical techniques are cost-effective and suitable for rapid monitoring with detecting multiple samples at the same time [50,51,52,53]. Within this category, ELISA is the most prevailing method [54,55,56,57,58,59,60,61]. Through the advancement of the immunoanalytical techniques, analytical sensitivities increased and LOD and LOQ values dropped to the same level as those for chromatographic methods. Among immunoanalytical methods, immunosensors represent innovative and more sensitive analytical determination techniques than microplate- or immunostrip-based detection [62,63,64,65,66,67,68].

Project Aquafluosense (NVKP_16-1-2016-0049) [69] aims to develop a new water analysis system for natural and artificial waters, allowing complex, systematic and in situ fluorescence-based assessment and monitoring of water quality. The modular instrument family developed for main parameters (chlorophyll-a content, chemical and biochemical oxygen demands, total organic carbon, polycyclic aromatic hydrocarbon, and certain agricultural pollutant contents) can be individually configured for target tasks at each monitoring point. Within the project, we aimed to develop an enzyme-linked fluorescent immunoassay (ELFIA) module for monitoring and quantification of ZON.

## 2. Results and Discussion

### 2.1. Determination of Zearalenone by Autofluorescence

**Figure 1:**
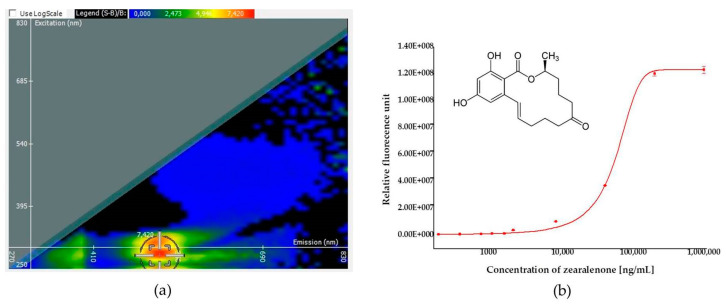
Results of zearalenone (ZON) quantification by autofluorescence. (**a**) A fluorescence spectral map of ZON in phosphate buffer saline and the optimized peak (in the range of a red patch, the middle point of cross-hair indicating optimal detection conditions) with 280 and 520 nm wavelengths for excitation (ordinate) and emission (abscissa), respectively. (**b**) A calibration curve obtained in a concentration range between 175 and 1,000,000 ng/mL of ZON and the chemical structure of ZON (insert).

Detection capability of ZON by its own induced fluorescence was assessed in direct fluorescence (autofluorescence) measurements in water. A fluorescence intensity spectral map and a calibration curve are presented on Figure 1. Fluorescence is generated by the optical excitation of electrons, which emit fluorescent light when they return to their ground state from their excited state. As a loss of vibrational energy inevitably occurs during this process, the emission spectrum is shifted to longer wavelengths than the excitation wavelength (Stokes shift). Figure 1 depicts the relevant wavelength pairs of excitation and emission of fluorescence spectra, as well as the dependence of intensity of the emitted light (fluorescence) on the concentration of ZON. Excitation mapping was carried out by scanning emission intensities as a function of excitation intensities between 250 and 830 nm wavelengths depicting emission intensity in a color scale from blue to red (Figure 1a). On the basis of the fluorescence spectral map, the optimized peak for ZON measurement by autofluorescence was obtained with excitation at 280 nm wavelength and emission detection at 520 nm wavelengths. The dependence of the emitted light at these parameters on the concentration of ZON in the aqueous sample was also tested and was found to follow a sigmoidal (logistic) regression (Figure 1b). Based on the sigmoid curve for autofluorescence, an LOD value of 11.5 µg/mL was determined.

### 2.2. Enzyme-Linked Fluorescent Immunoassay (ELFIA)

#### 2.2.1. Titration and Inhibition of the Antiserum

**Figure 2:**
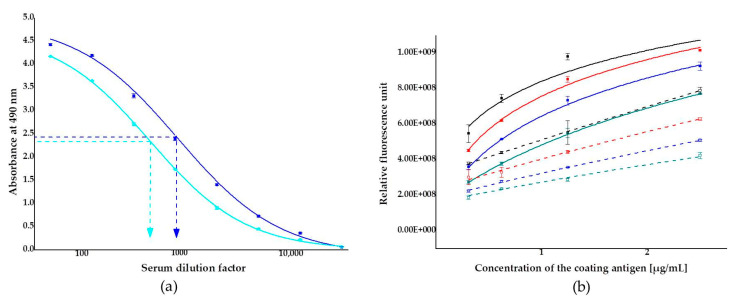
Analytical characterization of antisera collected from two 3-month old female New-Zealand white rabbits: (**a**) titer curves of antisera from rabbit 1 (■) and rabbit 2 (■) (dilution factor range of 1:50–1:12,200) using a zearalenone-6′-carboxymethyloxime-bovine serum albumin conjugate as a coating antigen at 5 µg/mL; blocked with 1% gelatin in phosphate buffer saline; (**b**) checkboard titration of the antiserum from rabbit 1 using the coating antigen at concentrations in the range of 0.3125–2.5 µg/mL and the serum at various dilution factors (solid symbols, solid lines): 1:1000 (■), 1:1500 (■), 1:2250 (■), 1:3375 (■). Titration was also performed under the same conditions with the serum inhibited by 3.2 ng/mL of zearalenone at various dilution factors (hollow symbols, slashed lines): 1:1000 (**☐**), 1:1500 (**☐**), 1:2250 (**☐**), and 1:3375 (**☐**).

Efficacy of the immunization was monitored by titration of the two rabbit antisera against the coating antigen, ZON conjugated to bovine serum albumin (BSA) (ZON–BSA) between 1:50 and 1:12,200 dilution factors. Microplates were coated with the BSA conjugate at concentrations of 1 to 5 µg/mL in coating buffer. Serum titers, defined as the serum dilution that binds 50% of the antigen under the given conditions, were determined for sera obtained from rabbits (rabbit 1 and rabbit 2). Only slight differences occurred between the efficacy of the two antisera: titer values were 1:828 and 1:448 for antisera from rabbit 1 and rabbit 2, respectively (Figure 2a). In the subsequent immunofluorescence assay experiments, antiserum from rabbit 1, showing somewhat higher affinity to the antigen, was applied. Accuracy and reproducibility of the measurements are better the nearer they are to the titer value, and thus antiserum was applied at a dilution factor of 1:1000 in the ELFIA tests.

To avoid the risk of saturatization or weak signal detection in assays, optimizations of the coating antigen concentration and serum dilution factor were performed by checkboard titration. The coating antigen, ZON-6′-carboxymethyloxime–BSA conjugate, was applied at concentrations in the range of 0.3125–2.5 µg/mL against the antiserum from rabbit 1 at dilutions in the range of 1:3375–1:1000 dilution factor. All combinations were investigated uninhibited and under inhibition by 3.2 ng/mL of ZON, as well. The coating antigen concentration and the antiserum dilution factor consistently influenced the analytical parameters (Figure 2b). The analytical signal (relative fluorescence unit, RFU) increased with increasing concentrations of the coating antigen and decreased with increasing dilution of the serum. The addition of ZON at a concentration of 3.2 ng/mL resulted in an average 40.0% ± 0.1% inhibition of the assay signal.

#### 2.2.2. Immunoassay

**Figure 3:**
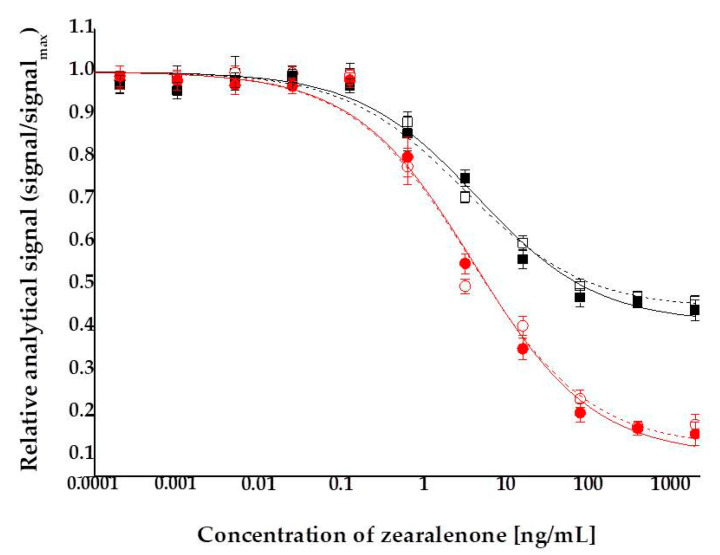
Competitive indirect calibration curves for zearalenone obtained in assay buffer (hollow marker, dashed lines) and in surface water from river Danube (solid marker, solid lines) determined by absorbance (■, □) and fluorescence (●, o), detected at 576 and 593 nm wavelengths, respectively.

Indirect competitive ELFIAs were performed to established ZON calibration curves and to determine the LOD. The detection range was investigated in a concentration series of 0.004 pg/mL–2 µg/mL ZON in assay buffer. Matrix effects were determined by comparing calibration curves obtained in assay buffer and in surface water samples. No significant differences were determined among curves (*p* > 0.05), thus it has been concluded that determination of ZON in surface water can be performed without modification in sample preparation. Calibration curves and LODs were determined using both absorbance and fluorescence signals (Figure 3). For comparability of the two detection modes, assay signals are represented as relative values (signals ratios to maximal signal levels). ZON at a concentration of 2000 ng/mL and above reached its full inhibition potential on the surface binding of the antibodies. This occurs because at this concentration the avidity of the primary antibody is saturated by ZON molecules in the solution, and therefore, further increases in ZON concentration cannot push the immunocomplexation equilibrium any further—ZON has reached its full capacity to block binding of the antibody to the coating antigen ZON–BSA conjugate on the surface of the immunoplate. The average relative analytical signal corresponding to the maximal assay signal produced by the uninhibited serum was set to the upper plateau level of the sigmoid standard curve. The average relative analytical signal corresponding to full inhibition of the serum was considered as the lower plateau level of the sigmoid standard curve. Analytical parameters of calibration curves are summarized in Table 1.

LOD values were calculated for the two analytical detection modes of resorufin as a chromophore product. Thus, LOD = 0.25 and 0.09 ng/mL were determined for visual absorbance and fluorescence detections, respectively. Detection by fluorescence provided a wider and steeper dynamic range, thus ELFIA proved to be a 2.8-fold more sensitive method for ZON than the corresponding ELISA. It has to be noted that absorbance detection of resorufin by the application of QuantaRed Enhanced Chemifluorescent HRP Substrate Kit with HRP enzyme reaction (Thermo Fisher Scientific Inc., Waltham, MA, USA) provided 3.4-fold lower LOD than that of o-phenylenediamine dihydrochloride (OPD) as chromophore in a similar colorimetric ELISA (LOD for OPD = 0.85 ng/mL).

#### 2.2.3. Effects of Light Source Intensity

Calibration curves were determined in the induced fluorescence method at different light source intensities. LED power can be digitally set between 0.001 µW and 4.63 mW in 256 nonequidistant steps at 532 nm wavelength, and it was tuned to provide assay signals between 20 and 30 RFU as the suggested least detectable value and 4095 RFU as the highest readable signal by the instrument. Correspondingly, the LED power values investigated ranged between 1.1 and 314 µW (1.1, 100.2, 169.4, 256 and 314 µW). As a background, 2000 ng/mL ZON solution was applied that triggered total inhibition of the antiserum. For 1.1 µW LED power there were no differences in the RFU values among different dilutions of ZON and for 314 µW LED power the RFU values reached the maximum (4095) at 0.64 ng/mL ZON concentration. Thus, effects of the LED power on ZON quantification were determined at values of 100.2, 169.4 and 256 µW. Recorded background signals were 24.7 ± 1.1, 27.9 ± 0.8 and 43.7 ± 1.0, while maximum (uninhibited) fluorescence levels were 1447, 2303 and 3131 for 100.2, 169.4, 256 µW, respectively. The analytical parameters of the calibration curves were determined with the optimized immunoassay system (see Section 4.4.3). In the data evaluation process, RFUs were corrected with the background. Among analytical parameters, IC_50_ values determined from the calibration curves in a concentration range of 0.0256–2000 ng/mL ZON were compared, and were found to be 2.52 ± 0.24, 3.04 ± 0.31 and 3.68 ± 0.29 ng/mL ZON for LED power values of 100.2, 169.4, 256 µW, respectively, while the LOD values did not appear to be significantly affected by the intensity of the light source.

#### 2.2.4. Cross-Reactivity of the Antisera with Zearalenone Derivatives

Inhibition of the antiserum by metabolites and structural analogues of ZON was also determined under optimized assay conditions. ZON is metabolized mostly through hydrolysis mainly to β- and α-zearalenol in yeast and ovine species, respectively [71,72]. Thus, IC_50_ values by ZON metabolites and their reduced derivatives (α- and β zearalenol, α- and β-zearalanol, zearalanone) were determined in the immunoassay by absorbance and fluorescence and relative cross-reactivities (considering inhibition by ZON as 100%) are listed in Table 2. The results indicate that the antibodies appear to be most sensitive to the presence of the unsaturation in the resorcyclic lactone ring and exhibit lower affinity to hydroxy metabolites. Stereoconfiguration of the hydroxyl group also occurs to influence recognition.

#### 2.2.5. Analytical Detection Capability Compared to Other Immunoanalytical Methods

The analytical performance of the above ELFIA method was compared to that of other immunoanalytical methods (immunoassays, immunosensors) reported in the scientific literature. Detection capabilities of the immunoanalytical methods are listed in Table 3. The immunoanalytical methods reported are mostly developed to be used for crop commodities (maize, wheat, barley, rice), and LODs are specified in the method descriptions as detectable ZON concentrations in the commodity (e.g., µg/kg). For this comparison, Table 3 enlists LODs in the final diluted extract according to the method specifications published. In these assays, organic solvent extracts in aqueous acetonitrile or methanol (MeOH) were used for ZON determination, but the solvent was diluted to 0.1% or below during dilution with the assay buffer to reach the detection range. LODs ranged between 10 ng/mL by an IgY-based ELISA [57] down to 0.002 pg/mL by an an optical waveguide ligthmode spectroscopy immunosensor [67]. As seen from the analytical sensitivity data, the current ELFIA method is located in the middle range of the reported procedures regarding the LOD values. Analytical detection performance can be possibly improved by using more specific antibodies, but the main advantage of the ELFIA method lies not primarily in its sensitivity, but in its utility in combined in situ application in the determination of other water quality parameters detectable by induced fluorimetry—e.g., total organic carbon content, algal density or other organic microcontaminants (herbicide glyphosate or pharmaceutical carbamazepin). In addition, the immunofluorescence module can be easily extended to other target analytes if proper antibodies are available for their detection.

### 2.3. High-Performance Liquid Chromatography (HPLC)

**Figure 4:**
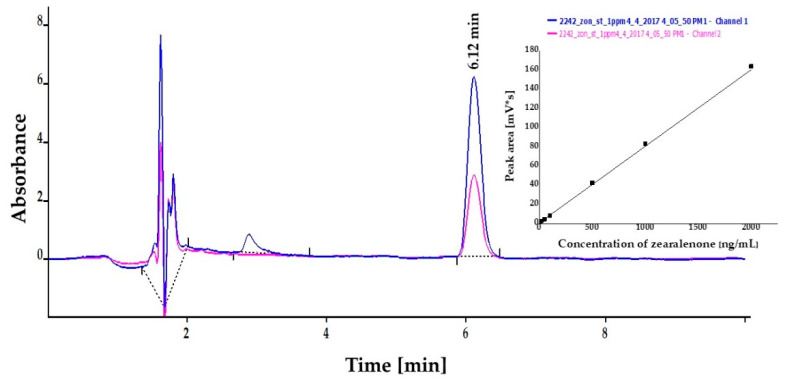
High-performance liquid chromatography (HPLC) chromatogram of zearalenone (ZON) at 1 µg/mL concentration dissolved in methanol:phosphate buffer saline (1:1). Linear calibration (average of peak area from three parallel measurements and their SDs) of ZON in a concentration range of 10–2000 ng/mL determined at 236 nm by high-performance liquid chromatography coupled with UV detection (insert).

Concentrations of ZON were also determined by HPLC instrumental analysis on the basis of peak areas in the chromatograms at the corresponding retention time (6.12 min) with excellent linear calibration characteristics in three parallel measurements. Peak areas determined at 236 nm for ZON concentrations between 10 and 2000 ng/mL were applied in linear regression, where regression coefficient of the concentration dependence was 0.999 in all measurements. Calibration curves with standard solutions were also investigated in MeOH:water = 7:3 and MeOH:phosphate buffer saline (PBS) = 1:1, where slope coefficients of linear regression were 26.90 ± 0.06 and 25.92 ± 0.06 for MeOH:water and MeOH:PBS, respectively. Peak purity was assessed by ratios of signal intensities (peak areas) recorded at 236 and 274 nm. These values for standard solutions were 2.15. Relative SDs established for different concentration levels for three parallel injections ranged between 0.65% and 1.76%. The LOD of the method was determined to be 10 ng/mL for ZON. A chromatogram and linear calibration (average of peak areas from three parallel measurements and their SDs) are presented in Figure 4.

### 2.4. Total Internal Reflection Ellipsometry

**Figure 5:**
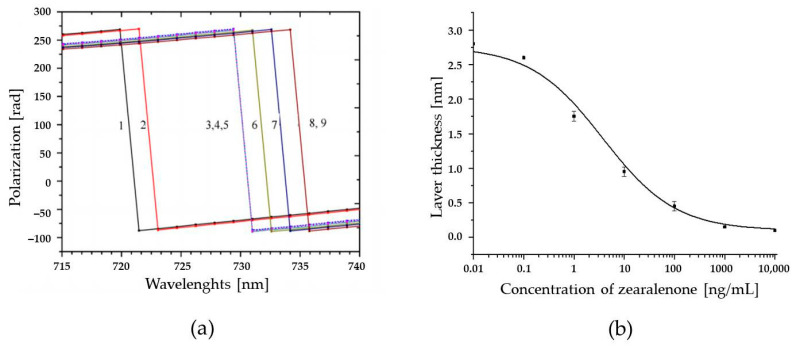
A competitive immunoassay for zearalenone (ZON) carried out by detection via total internal reflection ellipsometry (TIRE). (**a**) A typical set of Δ(λ) spectra measured on bare Au surface (1), polyallylamine hydrochloride (2) ZON–bovine serum albumin conjugate (3), bovine serum albumin (4), Ab-ZON of from preincubated mixtures containing ZON: 100 (5), 10 (6), 1 (7) and 0.1 ng/mL (8). (**b**) Changes in the adsorbed layer thickness versus the concentration of ZON (in the mixture with Ab-ZON) obtained by fitting the TIRE data.

The immunoassay setup has also been applied in a system based on detection by total internal reflection ellipsometry (TIRE), where two parameters Ψ and Δ are related, respectively, to the amplitude and phase shift of p- and s-components of the polarized light detected [73]. Since variations in the refractive index and the thickness of the adsorbed layers cause 10 times higher values of Δ than of Ψ, Δ(λ) spectra were used in the TIRE method as a sensor response. A typical series of Δ(λ) spectra for ZON competitive immunoassays and the respective calibration curve of the assay signal in TIRE (δd corresponding to the shift in the adsorbed layer thickness vs. the concentration of ZON) obtained by sigmoidal fitting are depicted in Figure 5. The response is similar to that shown in Figure 2, where the highest concentration of ZON yields the lowest response, which is typical for competitive immunoassays. On the basis of the standard calibration curve, a LOD of 0.01 ng/mL for ZON was determined.

## 3. Conclusions

Within project Aquafluosense, successful development resulted in a modular instrumentation setup for fluorescence-based determination of several characteristic parameters of water quality. The application of fluorescence, as an analytical signal in an enzyme-linked immunoassay format, results in a method of improved sensitivity with a lower LOD value than in the colorimetric ELISA (0.09 and 0.25 ng/mL, respectively). Moreover, resorufin-based determination proved lower LOD than application of OPD in colorimetric assay (LOD_OPD_ = 0.85 ng/mL). This benefit of this is that it allows determination of lower pollutant concentrations in surface water, which contributes to more effective monitoring. The detection results were validated by HPLC instrumental analysis and by a TIRE immunosensor method. Although the sensor technology provided orders of magnitude lower LOD than the immunofluorescence method developed, the great advantage of the latter is that it makes in situ determination possible, and the 96-well microplate format used in the immunofluorescence determination prototype allows an assay capacity of 25 samples in parallel in triplicates (with standard curves of seven calibration points). An in situ detection module with a dynamic detection range between 0.4 and 400 ng/mL was developed for ZON. The immunofluorescence method and the instrument prototype constitute a part of the Aquafluosense modular instrument family for determination of characteristic water parameters and contaminants.

## 4. Materials and Methods

### 4.1. Materials and Reagents

Organic chemicals and solvents, mycotoxin ZON and its derivatives, goat antirabbit immunoglobulin conjugated to horseradish peroxidase (HRP) as secondary antibody, and salts for buffers were purchased from Sigma-Aldrich Inc. (St. Louise, MO, USA). The purity of standard solutions was ≥98%. Immunoassays were carried out in high-capacity 96-well microplates (Nunc, Roskilde, Denmark) for colorimetric assay and in low profile 96-well microplates with white wells for increased fluorescence (Bio-Rad Laboratories, Hercules, CA, USA). QuantaRed Enhanced Chemifluorescent HRP Substrate Kit was used as the last step in immunoassays. Surface water samples were obtained from river Danube at Budapest and lakes Velencei at Agárd and Balaton at Tihany (sampling site GPS latitude and longitude and coordinates—Danube: 47.517519, 19.045519; lake Velencei: 47.200922, 18.578361; lake Balaton: 46.914043, 17.893401).

### 4.2. Instrumentation

**Figure 6:**
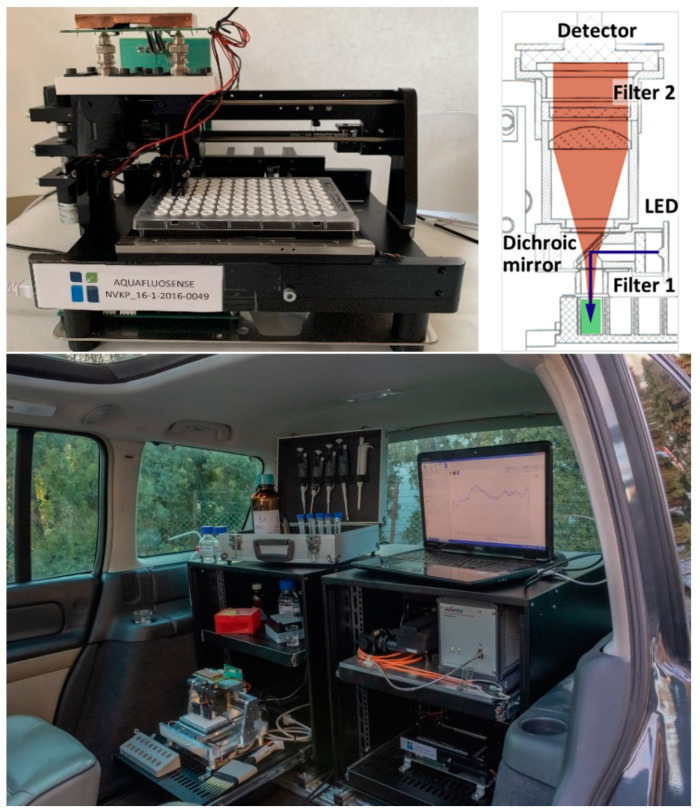
The immunofluorescence module developed in project Aquafluosense and appropriate for zearalenone determination (**top left**). The optical path in the detector head (**top right**). The modular instrumental setup during on site operation in a laboratory motor vehicle (**bottom**).

RFUs were determined by the prototype of a novel instrumentation developed in project Aquafluosense and realized in a modular setup (Figure 6). The instrument was developed partly (motor, optics, sample holder) at Optimal Optik Ltd. (Budapest, Hungary) and partly (detector electronics) at the Budapest University of Technology and Economics (Budapest, Hungary), and was designed to fit the 96-well ELISA microplate format using a self-designed, 3D-printed holder for ELFIA (Figure 6). The samples were illuminated in a dual head configuration with a high-power LED (Cree XPEBGR-L1-0000-00F01 with 520 to 535 nm minimum to maximum dominant wavelength range) in each head. The emitted fluorescence is measured in a dichroic beam path with silicon photodiodes (PIN-25D, OSI Optoelectronics) having large active area (d = 27.9 mm). The necessary high-spectral blocking and contrast was achieved by a combination of dichroic (Semrock FF562-Di03, edge: 562 nm) and bandpass optical filters on both the excitation (Semrock FF01-531/40-25, peak: 531 nm, width: 40 nm) and emission (Semrock FF01-593/40-25, peak: 593 nm, width: 40 nm) paths. The photodetector signal was coupled to a 2-stage amplifier unit (1st stage: OPA129 electrometer preamplifier, Texas Instruments; 2nd stage: AD620 instrumentation amplifier, Analog Devices) and then fed to a 12-bit analog-to-digital converter (Analog Devices AD7864-2 with 0 to 5 V unipolar input range) yielding 4095 resolvable RFUs. Gain and offset of the 2nd stage and the LED optical power were controlled by 256-stage (8-bit) digital potentiometers. As the LED power adjustment was nonequidistant, the optical power-control number curve was calibrated by a FieldMaxII-TO (Coherent) power meter with an OP-2 VIS sensor head set to a nominal wavelength of 532 nm. The instrument was equipped with stepping motors to move the detector heads over the 96-well microplates which provides fast and effective determination of individual RFUs in each microplate well. The instrument development is currently in the experimental phase; further decision about possible commercialization will be made by the project consortium.

### 4.3. Determination of Zearalenone by Autofluorescence

Fluorescence spectra of ZON were recorded on a SpectraMax iD3 Multi-Mode Microplate Reader (Molecular Devices, San Jose, CA, USA) by scanning the excitation wavelengths between 250 and 830 nm and emission wavelengths between 270 and 850 nm, with step sizes of 10 nm, where the emission wavelength must be a minimum of 20 nm greater than excitation. A spectrum map was established by RFUs measured for a solution of ZON in PBS at a concentration of 2000 ng/mL with a corresponding RFU for PBS at each point as a background. An optimized peak on the basis of the fluorescence spectral map obtained was applied to establish a calibration curve (in the concentration range of 0.6–2000 ng/mL) and a LOD of ZON based on autofluorescence.

### 4.4. Enzyme-Linked Immunofluorescence Assay

#### 4.4.1. Hapten Synthesis and Conjugation

The corresponding hapten, ZON-6′-carboxymethyloxime was converted from ZON by the method of Thouvenot and Morfin [61]. The reaction mixture containing 300 mg of ZON dissolved in dry pyridine and 600 mg of carboxymethoxylamine was stirred overnight at room temperature (22 ± 0.5 °C), then evaporated and the residue was taken up in 50 mL of slightly alkaline (pH = 8) water. The aqueous phase was extracted with ethyl acetate (4 × 100 mL). The organic phase was dried over sodium sulfate, then separated and evaporated to afford 156 mg of the product. The process of conversion of ZON to the corresponding hapten was followed by thin layer chromatography using hexane–ethyl acetate (1:2) as an eluent. BSA and conalbumin (CONA) were applied as carrier proteins. The hapten was conjugated to these proteins through amide bonds [55], using the active ester method for conjugation. Thus, 125 mg of the hapten ZON-6′-carboxymethyloxime was dissolved in 6.2 mL of dry tetrahydrofurane (THF), then 24 mg of *N*-hydroxy-succinimide and 73 mg of *N*,*N*′-dicyclohexylcarbodiimide were added. The mixture was stirred for 2 h at room temperature and the precipitation formed (dicyclohexylurea) was filtered off. In the mixture of 15.5 mL of water and 0.9 mL of THF, 150 mg of the proteins were dissolved in two separate batches. To these solutions, 3.1 mL of the above THF solution of the hapten active ester was added dropwise. The mixture was stirred for 24 h at 4 °C, and then the products (hapten–protein conjugate) were dialyzed against water at 4 °C for 1 week. Conjugation was monitored by UV spectroscopy; conjugates were lyophilized and stored at −20 °C until the analytical measurements.

#### 4.4.2. Serum Preparation

Two 3-month old female New Zealand white rabbits were immunized intradermally with the CONA-hapten conjugate (immunogen). Rabbit immunisation was performed under the supervision of the Ethics Committee of Research on Animals (Food Science Research Institute, Institute of Food Sciences, Hungarian University of Agriculture and Life Sciences, Budapest, Hungary) and under the authorisation and inspection by the Government Office for Pest County in Hungary (Official permit for animal testing # PE/EA/45-6/2020, last date of approval: 21 February 2020). Initial immunization was carried out with 0.1 mg immunogen dissolved in PBS and emulsified in Freund’s complete adjuvant (1:1 volume fraction). Then, injection of 0.15 mg of the immunogen in PBS and Freund’s incomplete adjuvant (1:1 volume fraction) was given (the first three booster injections at 3-week intervals, with the subsequent one at a 1-month interval). One week after each immunization, rabbits were bled and after coagulation of the blood (4 °C overnight) the sera were centrifuged (2400 g, 15 min) and purified by gel chromatography on PD-10 desalting columns.

#### 4.4.3. Immunoassay

Plates were coated with 1 µg/mL ZON-6′-carboxymethyloxime–BSA conjugate (ZON–BSA) in carbonate buffer (15 mM Na_2_CO_3_, 35 mM NaHCO_3_, pH = 9.6) overnight (~8 h) at 4 °C. The unbound conjugate was washed 4 times with PBS with 2% Tween20 (137 mM NaCl, 2.7 KCl, 10 mM Na_2_HPO_4_ × 2H_2_O, pH = 7.4). Blocking was carried out with 150 µL/well 1% gelatin in PBS, for 1.5 h of incubation at 37 °C. After washing, competition was initiated by adding 50 µL/well of both ZON analytical standard and purified rabbit anti-ZON serum (in PBS buffer with 5% Tween20, dilution 1:1000). An analytical ZON standard stock solution (1 mg/mL ZON in MeOH) was used in serial dilution (0.004 pg/mL–2 µg/mL). Thus, MeOH content in the actual ELFIA measurements was 0.2% or below. After 1 h of incubation at 37 °C and four washes, 100 µL/well goat antirabbit IgG–HRP (horseradish peroxidase, dilution 1:7500) conjugate was added as secondary antibody and incubated for 1 h at 37 °C. Unbound secondary antibodies were washed out 4 times with PBS and 100 µL/well working solution of QuantaRed Enhanced Chemifluorescent HRP Substrate Kit were added (content of the working solution: QuantaRed ADHP concentrate, QuantaRed Enhancer Solution, and QuantaRed stable Peroxide in 1:50:50 (*v*/*v*) proportion). The kit contains 10-acetyl-3,7-dihydroxyphenoxazine (ADHP), a nonfluorescent compound that is dehydrogenated (oxidized) by HRP to resorufin, a highly fluorescent reaction product, which can also be measured in a colorimetric plate reader. After 5 min of incubation, the enzymatic activity was stopped by 10 µL of QuantaRed Stop Solution. Both absorbance and fluorescence were measured at 576 and at 593 nm wavelengths, respectively. After the colorimetric assay, the liquid phase was transferred with an 8-channel pipette into a white, low profile 96-well microplate where fluorescence was determined. Absorbances were read by SpectraMax iD3 Multi-Mode Microplate Reader (Molecular Devices, San Jose, CA, USA) at 576 nm wavelength, while relative fluorescence signals were determined by the prototype fluorimeter developed in project Aquafluosense [69,74] (see Section 4.2). Standard curves were obtained in different surface water samples as well, for evaluation of matrix effects in ZON determination from natural water bodies. Statistical analysis of standard curves was performed by comparison of IC_50_ values by one-way analysis of variance followed by post hoc Tukey test at a significance level of 0.05.

The immunoassay developed in this study can be performed in situ by the instrument developed and built in a laboratory motor vehicle. Coating the assay microplates by the ZON–BSA conjugate can be carried out under laboratory conditions (method in Section 4.4.3) prior the measurement, and in situ determination can be performed on precoated microplates in the mobile equipment. Thus, the total immunoassay performance time is 2.5 h. Since the immunofluorescence instrument is equipped with stepping motor units, fluorescence determination on an entire plate requires approximately 2 min. A possibility of further shortening the procedure is potentially through performing both the coating and blocking steps in the laboratory a day before the in situ determination. In this case, the coating step can be applied at room temperature for 1 h and the blocking step with 1% gelatin in PBS for 1.5 h. Coated and blocked microplates can be stored at 4 °C until measurement; however, it is necessary to prevent evaporation in the wells by covering the microplate with parafilm.

### 4.5. High-Performance Liquid Chromatography (HPLC)

A calibration curve and the LOD for ZON were determined also by HPLC measurements on a Younglin YL9100 HPLC system equipped with a YL9150 autosampler (YL Instruments, Anyang, Korea). Compounds were separated on a column (150 × 4.6 mm i.d., 5 μm) at 30 °C containing C18 stationary phase. The PerfectSil 100 ODS-3 column was manufactured by MZ-Analysentechnik GmbH (Mainz, Germany). UV detector signals were recorded at *λ*_1_ = 236 nm and *λ*_2_ = 274 nm. Eluent flow rate was 1.0 mL/min with isocratic elution for 10 min (30:70 = A:B eluents, A = 90% water:10% MeOH, B = MeOH). The retention time of ZON under the current conditions was 6.12 min. LOD, defined as an analyte concentration corresponding to a signal level of signal/noise ratio of 3, was determined with standard solutions. For establishing an analytical standard curve, a stock solution of ZON at a concentration of 1.0 g/mL was prepared in MeOH. Calibration curves were obtained with 7 standard solutions between 10 and 2000 ng/mL in MeOH:water = 7:3) and MeOH:PBS (1:1).

### 4.6. Total Internal Reflection Ellipsometry (TIRE)

Total internal reflection ellipsometry (TIRE) experiments were carried out on an M-2000 automatic spectroscopic ellipsometer (J.A. Woollam Co., Lincoln, NE, USA) operating in the 370–1000 nm range using glass-based sensor chips fabricated in the laboratory by vacuum evaporation. Sensor surfaces were prepared by thermal evaporation of layers of chromium (Cr)—3 nm thick and gold (Au)—25 nm on standard microscopic glass slides (BK-7). The Cr layer improves the adhesion of gold to the glass surface. The Au surface was modified with mercaptoethyl sodium sulfonate to enhance the negative surface charge. The ellipsometer was equipped with a 68° trapezoidal prism which allowed coupling the light beam at total internal reflection conditions to the gold film on the glass slide. The 0.2 mL reaction cell with the inlet and outlet tubes was attached underneath to the gold surface, allowing the injection of the required chemicals to perform binding reactions. The ellipsometry spectral scans were performed in a standard Trisma/HCl buffer solution (pH = 7.5) after completing each adsorption (binding) stage. For a competitive immunoassay, a ZON-6′-carboxymethyloxime–BSA conjugate (ZON–BSA) was electrostatically immobilized on the Au surface via a polyallylamine hydrochloride layer. In order to block all the remaining binding sites, an additional adsorption of BSA was carried out. Then a mixture of ZON-specific antiserum and solutions of free ZON (at a concentration range of 0.01 ng/mL–10 µg/mL) were injected into the cell with the intermediate rinsing with buffer. The mixtures were preincubated for 5 min before injection. A series of Δ spectra were recorded after binding of ZON to the antibodies immobilized on the chip surface.

## Figures and Tables

**Table 1:** Statistical parameters of the logistic mathematical fitting using the Rodbard equation [70] in the immunoassay format using assay signals by resorufin as a chromophore product with detection of absorbance and fluorescence.

**Equation for fitting:** $y=\frac{A_{1}-A_{2}}{1+{\left(\frac{x}{x_{0}}\right)}^{p}}+A_{2}$ ^1^		
Adjusted R^2^: 0.990 (absorbance) 0.988 (fluorescence)		
	**Parameter**	**Value ± Standard Deviation**
Absorbance	$A_{1}$	0.98 ± 0.02
	$A_{2}$	0.45 ± 0.01
	$x_{0}$	2.86 ± 0.38
	p	0.79 ± 0.12
Fluorescence	$A_{1}$	0.99 ± 0.01
	$A_{2}$	0.16 ± 0.03
	$x_{0}$	2.41 ± 0.27
	p	0.83 ± 0.19

^1^ Description of the equation parameters—$A_{1}$: upper plateau, $A_{2}$: lower plateau, $x_{0}$: 50% inhibition, *p*: curve slope at the inflexion (IC_50_).

**Table 2:** Percentage cross-reactivity (CR%) of the antiserum with zearalenone and its derivatives determined by fluorescence and absorbance.

Mycotoxin	Detection Mode			
	Fluorescence		Absorbance	
	IC_50_ (ng/mL) ^1^	CR% ^2^	IC_50_ (ng/mL)	CR% ^1^
zearalenone	2.20 ± 0.31	100	2.73 ± 0.35	100
α-zearalenol	10.42 ± 0.24	21.1 ± 3.0	10.94 ± 1.28	20.1 ± 2.6
β-zearalenol	8.74 ± 0.90	25.2 ± 3.6	8.65 ± 0.84	25.4 ± 3.3
zearalanone	8.56 ± 0.74	25.7 ± 3.6	8.24 ± 0.82	26.7 ± 3.4
α-zearalanol	35.36 ± 2.86	6.2 ± 0.9	35.63 ± 3.05	6.2 ± 0.8
β-zearalanol	200.7 ± 12.32	1.1 ± 0.2	250.02 ± 21.68	0.9 ± 0.1

^1^ IC_50_: half maximal inhibitory concentration; ^2^ CR%: Cross-reactivity defined as the percentage ratio of the IC_50_ values of zearalenone and of the given derivative.

**Table 3:** Analytical performance characteristics of various immunoanalytical methods for the determination of zearalenone.

Analytical Method	LOD ^1^ (ng/mL)	IC_50_^2^ *Detection Range* (ng/mL)	Matrix	Organic Solvent Content in the Sample Extract	Reference
ELISA ^3^	10	40 *10–200*	maize	10% AcCN ^4^	[57]
Radioimmunoassay	5	NR ^5^ *0.25–10*	human serum	-	[61]
ELISA	1	3 *0.5–50*	wheat, maize	10% MeOH ^6^	[55]
SPR (Sensor) ^7^	0.56	5	wheat	16% MeOH	[65]
DPV (Sensor) ^8^	0.25	NR	beer, wine	20% AcCN	[64]
ELISA	0.24	0.855 ^9^	maize	14% MeOH	[54]
ELISA	0.15 (PBS) 0.23 (maize)	1.13 (PBS) 1.4 (maize)	maize	8% MeOH	[60]
FLISA ^10^	0.10	0.95	maize flour	14% MeOH	[56]
ELFIA ^11^	0.09	2.4	water	0.2% MeOH	this study
ELISA	0.05	NR	wheat	10% MeOH	[58]
ELISA	0.02	0.18	maize flour	14% MeOH	[56]
SPR (Sensor)	0.01	NR	NR	9% AcCN	[63]
PW PI (Sensor) ^12^	0.01	NR	water	10% MeOH	[66]
CPG-Based Immunosensor ^13^	0.007	0.087	wheat	0.21% MeOH; 0.2% AcCN	[68]
ELISA (Coupled with IAC) ^14^	0.002	0.02	maize	10% AcCN	[59]
OWLS (Sensor) ^15^	2 × 10^−6^	0.014	maize	AcCN	[54]

^1^ LOD: limit of detection; ^2^ IC_50_: half maximal inhibitory concentration; ^3^ ELISA: enzyme-linked immunosorbent assay; ^4^ AcCN: acetonitrile; ^5^ NR: not reported; ^6^ MeOH: methanol; ^7^ SPR: surface plasmon resonance; ^8^ DPV: differential pulse voltammetry; ^9^ PBS: phosphate buffer saline; ^10^ FLISA: fluorescence-linked immunosorbent assay; ^11^ ELFIA: enzyme-linked fluorescent immunoassay; ^12^ PW PI: planar waveguide operating as a polarization interferometer; ^13^ CPG: controlled pore glass; ^14^ IAC: immunoaffinity column; ^15^ OWLS: optical waveguide lightmode spectroscopy.

## Data Availability

The data presented in this study are available on request from the corresponding author. The data are not publicly available due to privacy reasons.

## References

[1] Khaneghah A.M., Fakhri Y., Gahruie H.H., Niakousari M., Sant’Ana A.S. (2019). Mycotoxins in cereal-based products during 24 years (1983–2017): A global systematic review. Trends Food Sci. Technol..

[2] Cimbalo A., Alonso-Garrido M., Font G., Manyes L. (2020). Toxicity of mycotoxins in vivo on vertebrate organisms: A review. Food Chem. Toxicol..

[3] Krska R., Molinelli A. (2006). Mycotoxin analysis: State-of-the-art and future trends. Anal. Bioanal. Chem..

[4] Rubert J., Soriano J.M., Mañes J., Soler C. (2011). Rapid mycotoxin analysis in human urine: A pilot study. Food Chem. Toxicol..

[5] Van Der Fels-Klerx H., De Rijk T., Booij C., Goedhart P., Boers E., Zhao C., Waalwijk C., Mol H., Van Der Lee T. (2012). Occurrence of *Fusarium* head blight species and *Fusarium* mycotoxins in winter wheat in the Netherlands in 2009. Food Addit. Contam. Part A.

[6] Valverde-Bogantes E., Bianchini A., Herr J.R., Rose D.J., Wegulo S.N., Hallen-Adams H.E. (2020). Recent population changes of *Fusarium* head blight pathogens: Drivers and implications. Can. J. Plant Pathol..

[7] Valencia-Quintana R., Milić M., Jakšić D., Šegvić Klarić M., Tenorio-Arvide M.G., Pérez-Flores G.A., Bonassi S., Sánchez-Alarcón J. (2020). Environment changes, aflatoxins, and health issues, a review. Int. J. Environ. Res. Public Health.

[8] Dänicke S., Winkler J. (2015). Invited review: Diagnosis of zearalenone (ZEN) exposure of farm animals and transfer of its residues into edible tissues (carry over). Food Chem. Toxicol..

[9] Zheng W., Feng N., Wang Y., Noll L., Xu S., Liu X., Lu N., Zou H., Gu J., Yuan Y. (2019). Effects of zearalenone and its derivatives on the synthesis and secretion of mammalian sex steroid hormones: A review. Food Chem. Toxicol..

[10] Kelley J., Paterson R.R.M., Lia N., Smith D. (2003). Comparisons of ergosterol to other methods for determination of *Fusarium graminearum* biomass in water as a model system. Proceedings of the 22nd European Culture Collections’ Organization Meeting.

[11] Picardo M., Filatova D., Nuñez O., Farré M. (2019). Recent advances in the detection of natural toxins in freshwater environments. TrAC Trends Anal. Chem..

[12] Russell R., Paterson M. (2007). Zearalenone production and growth in drinking water inoculated with *Fusarium graminearum*. Mycol. Prog..

[13] Bucheli T.D., Wettstein F.E., Hartmann N., Erbs M., Vogelgsang S., Forrer H.-R., Schwarzenbach R.P. (2008). *Fusarium mycotoxins*: Overlooked aquatic micropollutants?. J. Agric. Food Chem..

[14] Gromadzka K., Waśkiewicz A., Goliński P., Świetlik J. (2009). Occurrence of estrogenic mycotoxin—Zearalenone in aqueous environmental samples with various NOM content. Water Res..

[15] Waśkiewicz A., Gromadzka K., Bocianowski J., Pluta P., Goliński P. (2012). Zearalenone Contamination of the aquatic environment as a result of its presence in crops/Pojava mikotoksina u vodenom okolišu zbog njihove prisutnosti u usjevima. Arch. Ind. Hyg. Toxicol..

[16] Jarošová B., Javůrek J., Adamovský O., Hilscherová K. (2015). Phytoestrogens and mycoestrogens in surface waters—Their sources, occurrence, and potential contribution to estrogenic activity. Environ. Int..

[17] Laranjeiro C.S., Da Silva L.J.G., Pereira A.M., Pena A., Lino C.M. (2017). The mycoestrogen zearalenone in Portuguese flowing waters and its potential environmental impact. Mycotoxin Res..

[18] Al-Gabr H.M., Zheng T., Yu X. (2013). Fungi contamination of drinking water. Rev. Environ. Contam. Toxicol..

[19] Hartmann N., Erbs M., Wettstein F.E., Schwarzenbach R.P., Bucheli T.D. (2007). Quantification of estrogenic mycotoxins at the ng/L level in aqueous environmental samples using deuterated internal standards. J. Chromatogr. A.

[20] Criado M.V., Pinto V.E.F., Badessari A., Cabral D. (2005). Conditions that regulate the growth of moulds inoculated into bottled mineral water. Int. J. Food Microbiol..

[21] Pereira V., Fernandes D., Carvalho G., Benoliel M., Romão M.S., Crespo M.B. (2010). Assessment of the presence and dynamics of fungi in drinking water sources using cultural and molecular methods. Water Res..

[22] Kolpin D.W., Schenzel J., Meyer M.T., Phillips P.J., Hubbard L.E., Scott T.-M., Bucheli T.D. (2014). Mycotoxins: Diffuse and point source contributions of natural contaminants of emerging concern to streams. Sci. Total. Environ..

[23] Babič M.N., Gunde-Cimerman N., Vargha M., Tischner Z., Magyar D., Veríssimo C., Sabino R., Viegas C., Meyer W., Brandão J. (2017). Fungal contaminants in drinking water regulation? A tale of ecology, exposure, purification and clinical relevance. Int. J. Environ. Res. Public Health.

[24] Paterson R., Kelley J., Gallagher M. (1997). Natural occurrence of aflatoxins and *Aspergillus flaws* (Link) in water. Lett. Appl. Microbiol..

[25] Oliveira B.R., Mata A.T., Ferreira J.P., Crespo M.T.B., Pereira V.J., Bronze M.R. (2018). Production of mycotoxins by filamentous fungi in untreated surface water. Environ. Sci. Pollut. Res..

[26] Kolpin D.W., Hoerger C.C., Meyer M.T., Wettstein F.E., Hubbard L.E., Bucheli T.D. (2010). Phytoestrogens and Mycotoxins in Iowa Streams: An examination of underinvestigated compounds in agricultural basins. J. Environ. Qual..

[27] Mata A., Ferreira J., Oliveira B., Batoréu M., Crespo M.B., Pereira V., Bronze M. (2015). Bottled water: Analysis of mycotoxins by LC–MS/MS. Food Chem..

[28] Waśkiewicz A., Bocianowski J., Perczak A., Goliński P. (2015). Occurrence of fungal metabolites—Fumonisins at the ng/L level in aqueous environmental samples. Sci. Total. Environ..

[29] Emídio E.S., Da Silva C.P., De Marchi M.R.R. (2020). Estrogenic mycotoxins in surface waters of the Rico Stream micro-basin, São Paulo, Brazil: Occurrence and potential estrogenic contribution. Eclética Química J..

[30] Maragos C.M. (2012). Zearalenone occurrence in surface waters in central Illinois, USA. Food Addit. Contam. Part B.

[31] Hartmann N., Erbs M., Forrer H.-R., Vogelgsang S., Wettstein F.E., Schwarzenbach R.P., Bucheli T.D. (2008). Occurrence of Zearalenone on *Fusarium graminearum* infected wheat and maize fields in crop organs, soil, and drainage water. Environ. Sci. Technol..

[32] Laganà A., Bacaloni A., De Leva I., Faberi A., Fago G., Marino A. (2004). Analytical methodologies for determining the occurrence of endocrine disrupting chemicals in sewage treatment plants and natural waters. Anal. Chim. Acta.

[33] Pietsch C. (2017). Zearalenone (ZEN) and its influence on regulation of gene expression in carp (*Cyprinus carpio* L.) liver tissue. Toxins.

[34] Khezri A., Herranz-Jusdado J.G., Ropstad E., Fraser T.W. (2018). Mycotoxins induce developmental toxicity and behavioural aberrations in zebrafish larvae. Environ. Pollut..

[35] European Food Safety Authority (EFSA) (2011). Scientific Opinion on the risks for public health related to the presence of zearalenone in food. EFSA J..

[36] European Commission (2006). Commission regulation (EC) No 1881/2006 of 19 December 2006. Setting maximum levels for certain contaminants in foodstuffs. Off. J. Eur. Union.

[37] EFSA Panel on Contaminants in the Food Chain (CONTAM) (2014). Scientific Opinion on the risks for human and animal health related to the presence of modified forms of certain mycotoxins in food and feed. EFSA J..

[38] Knutsen H., Alexander J., Barregård L., Bignami M., Brüschweiler B., Ceccatelli S., Cottrill B., DiNovi M., Edler L., EFSA Panel on Contaminants in the Food Chain (CONTAM) (2017). Risks for animal health related to the presence of zearalenone and its modified forms in feed. EFSA J..

[39] Caglayan M.O., Şahin S., Üstündağ Z. (2020). Detection strategies of zearalenone for food safety: A review. Crit. Rev. Anal. Chem..

[40] Turner G.V., Phillips T.D., Heidelbaugh N.D., Russell L.H. (1983). High Pressure liquid chromatographic determination of zearalenone in chicken tissues. J. Assoc. Off. Anal. Chem..

[41] Sebaei A.S., Gomaa A.M., Mohamed G.G., El-Di F.N. (2012). Simple validated method for determination of deoxynivalenol and zearalenone in some cereals using high performance liquid chromatography. Am. J. Food Technol..

[42] Majerus P., Graf N., Krämer M. (2009). Rapid determination of zearalenone in edible oils by HPLC with fluorescence detection. Mycotoxin Res..

[43] Scott P.M., Lawrence J.W., van Walbeek W. (1970). Detection of mycotoxins by thin-layer chromatography: Application to screening of fungal extracts. App. Environ. Microbiol..

[44] Larionova D.A., Goryacheva I.Y., Van Peteghem C., De Saeger S. (2011). Thin-layer chromatography of aflatoxins and zearalenones with β-cyclodextrins as mobile phase additives. World Mycotoxin J..

[45] Soares L.M.V. (1992). Multi-toxin TLC methods for aflatoxins, ochratoxin a, zearalenone and sterigmatocystin in foods. Analysis of Plant Waste Materials.

[46] Thouvenot D., Morfin R. (1979). Quantitation of zearalenone by gas-liquid chromatography on capillary glass columns. J. Chromatogr. A.

[47] Yan Z., Wang L., Wang J., Tan Y., Yu D., Chang X., Fan Y., Zhao D., Wang C., De Boevre M. (2018). A QuEChERS-based liquid chromatography-tandem mass spectrometry method for the simultaneous determination of nine zearalenone-like mycotoxins in pigs. Toxins.

[48] De Santis B., Debegnach F., Gregori E., Russo S., Marchegiani F., Moracci G., Brera C. (2017). Development of a LC-MS/MS Method for the multi-mycotoxin determination in composite cereal-based samples. Toxins.

[49] Jodlbauer J., Zöllner P., Lindner W. (2000). Determination of zeranol, taleranol, zearalenone, α- and β-zearalenol in urine and tissue by high-performance liquid chromatography-tandem mass spectrometry. Chromatographia.

[50] Pestka J.J., Abouzied M.N. (1995). Immunological assays for mycotoxin detection. Food Technol..

[51] Maragos C.M., Kim E.-K. (2004). Detection of zearalenone and related metabolites by fluorescence polarization immunoassay. J. Food Prot..

[52] Hao K., Suryoprabowo S., Song S., Liu L., Kuang H. (2017). Rapid detection of zearalenone and its metabolite in corn flour with the immunochromatographic test strip. Food Agric. Immunol..

[53] Renaud J.B., Miller J.D., Sumarah M.W. (2019). Mycotoxin testing paradigm: Challenges and opportunities for the future. J. AOAC Int..

[54] Dong G., Pan Y., Wang Y., Ahmed S., Liu Z., Peng D., Yuan Z. (2018). Preparation of a broad-spectrum anti-zearalenone and its primary analogues antibody and its application in an indirect competitive enzyme-linked immunosorbent assay. Food Chem..

[55] Liu M.T., Ram B.P., Hart L.P., Pestka J.J. (1985). Indirect enzyme-linked immunosorbent assay for the mycotoxin zearalenone. Appl. Environ. Microbiol..

[56] Liu N., Nie D., Zhao Z., Meng X., Wu A. (2015). Ultrasensitive immunoassays based on biotin–streptavidin amplified system for quantitative determination of family zearalenones. Food Control..

[57] Pichler H., Krska R., Szekacs A., Grasserbauer M. (1998). An enzyme-immunoassay for the detection of the mycotoxin zearalenone by use of yolk antibodies. Anal. Bioanal. Chem..

[58] Radová Z., HajšLová J., Králová J., Papoušková L., Sýkorová S. (2001). Analysis of Zearalenone in wheat using high-performance liquid chromatography with fluorescence detection and/or enzyme-linked immunosorbent assay. Cereal Res. Commun..

[59] Tang X., Li X., Li P., Zhang Q., Li R., Zhang W., Ding X., Lei J., Zhang Z. (2014). Development and application of an immu-noaffinity column enzyme immunoassay for mycotoxin zearalenone in complicated samples. PLoS ONE.

[60] Thongrussamee T., Kuzmina N., Shim W.-B., Jiratpong T., Eremin S., Intrasook J., Chung D.-H. (2008). Monoclonal-based enzyme-linked immunosorbent assay for the detection of zearalenone in cereals. Food Addit. Contam. Part A.

[61] Thouvenot D., Morfin R.F. (1983). Radioimmunoassay for zearalenone and zearalanol in human serum: Production, properties, and use of porcine antibodies. Appl. Environ. Microbiol..

[62] Chun H.S., Choi E.H., Chang H.-J., Choi S.-W., Eremin S.A. (2009). A fluorescence polarization immunoassay for the detection of zearalenone in corn. Anal. Chim. Acta.

[63] Van Der Gaag B., Spath S., Dietrich H., Stigter E., Boonzaaijer G., Van Osenbruggen T., Koopal K. (2003). Biosensors and multiple mycotoxin analysis. Food Control..

[64] Goud Y.K., Kumar S.V., Hayat K., Gobi V.K., Song H., Kim K.-H., Marty J.L. (2019). A highly sensitive electrochemical immunosensor for zearalenone using screen-printed disposable electrodes. J. Electroanal. Chem..

[65] Hossain M.Z., Maragos C.M. (2018). Gold nanoparticle-enhanced multiplexed imaging surface plasmon resonance (iSPR) detection of *Fusarium* mycotoxins in wheat. Biosens. Bioelectron..

[66] Nabok A., Al-Jawdah A., Gémes B., Takács E., Székács A. (2021). An optical planar waveguide-based immunosensors for determination of *Fusarium* mycotoxin zearalenone. Toxins.

[67] Székács I., Adányi N., Szendrő I., Székács A. (2021). Direct and Competitive optical grating immunosensors for determination of *Fusarium* mycotoxin zearalenone. Toxins.

[68] Urraca J.L., Benito-Peña E., Pérez-Conde C., Moreno-Bondi M.C., Pestka J.J. (2005). Analysis of zearalenone in cereal and swine feed samples using an automated flow-through immunosensor. J. Agric. Food Chem..

[69] Aquafluosense. komplex vízminősítést in situ megvalósító, közvetlen és immunfluoreszcencián, valamint optikai és lézeres plazma-színképelemzésen alapuló, moduláris, érzékelő- és műszercsalád kifejlesztése, továbbá az alkalmazási területek kutatása. http://aquafluosense.hu.

[70] Rodbard D., Hutt D.M. Statistical analysis of radioimmunoassays and immunoradiometric (labeled antibody) assays: A generalized, weighted, iterative, least-squares method for logistic curve fitting. Proceedings of the Symposium on RIA and Related Procedures in Medicine, Proceedings of the Internationaé Atomic Energy Agency.

[71] Miles C.O., Erasmuson A.F., Wilkins A.L., Towers N.R., Smith B.L., Garthwaite I., Scahill B.G., Hansen R.P. (1996). Ovine metabolism of zearalenone to α-zearalanol (Zeranol). J. Agric. Food Chem..

[72] Megharaj M., Garthwaite I., Thiele J.H. (1997). Total biodegradation of the oestrogenic mycotoxin zearalenone by a bacterial culture. Lett. Appl. Microbiol..

[73] Nabok A., Tsargorodskaya A. (2008). The method of total internal reflection ellipsometry for thin film characterisation and sensing. Thin Solid Film..

[74] Csősz D., Lenk S., Barócsi A., Csőke T.L., Klátyik S., Lázár D., Berki M., Adányi N., Csákányi A., Domján L. (2019). Sensitive fluorescence instrumentation for water quality assessment. Proceedings of the Optical Sensors and Sensing Congress (ES, FTS, HISE, Sensors).

